# Decline in Pediatric Shelf Examination Performance During COVID-19

**DOI:** 10.7759/cureus.18453

**Published:** 2021-10-03

**Authors:** Amy E Hanson, April P'Pool, Michelle C Starr, Bobbi J Byrne

**Affiliations:** 1 Department of Pediatrics, Indiana University School of Medicine, Indianapolis, USA; 2 Department of Pediatrics, Division of Pediatric Nephrology, Indiana University School of Medicine, Indianapolis, USA

**Keywords:** medical student exams, medical student grading, nbme shelf, covid-19 medical education, pediatric clerkship, pediatrics, covid-19

## Abstract

Background

Medical student education has been impacted by the ongoing coronavirus 2019 (COVID-19) pandemic. Medical students were removed from clinical settings, and the censuses in pediatric hospitals decreased. While there have been studies starting to evaluate these effects on medical students training in surgical subspecialties, the literature in pediatrics is limited.

Objective

This study analyzed third-year medical students’ National Board of Medical Examiners (NBME) Clinical Science Pediatrics Shelf Exam scores at the conclusion of their core pediatric clerkship. We compared the exam scores before COVID-19 pandemic to those during the pandemic. We hypothesized that the ongoing COVID-19 pandemic would have a negative impact on NBME shelf exam scores and that shelf exam failure rates would increase.

Methods

Institutional Review Board approval was obtained prior to initiation of this study. We conducted a retrospective review of medical student pediatric shelf exam scores from June 2017 to December 2020 from one large, single institution. We adjusted scores for block schedule timing and standardized them based on national norms published for the year prior. We compared two groups: those who completed their pediatric clerkship experiences before pandemic (predominantly in-person learning) vs. those who completed it during the pandemic (predominantly virtual learning). Groups were compared using chi-square and analysis-of-variance testing.

Results

We included 991 medical students, 772 before COVID-19 and 219 during COVID-19. Of these, 19 of 772 (2.5%) students failed the exam prior to COVID-19 compared to 19 of 219 (8.7%) during COVID-19 (*p* < 0.001). Students who completed their pediatric clerkship during COVID-19 were 3.77 times more likely to fail their end-of-clerkship NBME shelf exam (*p* < 0.001).

Conclusions

Students who completed their core pediatric clerkship in a predominantly virtual platform during the COVID-19 pandemic were significantly more likely to fail their end-of-clerkship NBME shelf exam. Increased failure rates may suggest issues with acquisition and retainment of pediatric medical knowledge throughout the clerkship, creating knowledge gaps in the foundation of their pediatric experience. Long-term effects of virtual learning platforms will need to be studied further.

## Introduction

Medical student education has been drastically altered world-wide since the onset of the novel coronavirus 2019 (COVID-19) pandemic in December of 2019 [1-6]. Traditionally at our United States (US)-based institution, Indiana University School of Medicine (IUSOM), medical students completed two years of didactic curriculum followed by completion of their United States Medical Licensing Exam (USMLE) Step 1 exam during spring of the second year [6]. Upon passage of Step 1, students continue into the core clerkship year.

During the COVID-19 pandemic, these practices changed at IUSOM. In March 2020, IUSOM suspended medical students from clinical experiences and converted immediately to virtual curriculum. Prometric testing centers closed their doors, requiring medical students to postpone their Step 1 exams indefinitely. These changes were followed by Association of American Medical Colleges (AAMC)'s recommendations released on March 17, 2020, that all US medical schools suspend clinical clerkships for at least two weeks [3-7]. These recommendations were quickly extended to April 14, 2020 and reported that AAMC “strongly suggest[s] that medical students not be involved in any direct patient care activities” [6]. In July 2020, IUSOM initiated an online education lecture series for each of the core clerkships. This was quickly followed by transition back into clinical settings for students in August 2020. Given five months of lost clerkship time, the pediatric core clerkship was condensed from its original 8 weeks (4 weeks inpatient, 4 weeks outpatient) to 5-6 weeks (approximately 2.5 weeks inpatient, 2.5 weeks outpatient).

Outpatient clinic experience became limited due to rapid transition to telehealth. Outpatient clinic time often became independent study time. Inpatient exposure was complicated by an increase in the number of students rotating on each clinical service at any given time and overall decreased hospital census. Additionally, students were prohibited from caring for potentially COVID-19-positive patients. Students, however, were still required to take their end-of-clerkship National Board of Medical Examiners (NBME) Clinical Science Pediatrics Shelf Exams.

This study aimed to evaluate whether shelf scores were affected by the changes described above in the context of the COVID-19 pandemic. There have not been previous publications assessing this question in the pediatric literature. We hypothesized that NBME shelf exam failure rates would increase during the COVID-19 pandemic.

## Materials and methods

We performed a retrospective review of medical student pediatric clerkship shelf exam scores from one large, single institution. IUSOM is the largest MD-granting medical school in the US, educating over 1,450 medical students across nine statewide campuses [8]. Shelf exam scores available for students who completed their core pediatric clerkship at the main medical campus in Indianapolis, IN, between June 2017 and December 2020 were obtained (n = 991). Pediatric clerkship coordinators provided the authors with access to score reports sorted by testing block via shared, dual-authentication password-secured IUSOM Box storage. Authors then de-identified the scores by entering them into a password-protected Microsoft Excel Version 2018 file, where they were re-assigned to a randomized identification number but remained paired with their block dates.

These scores were standardized to national percentiles by quarter based on the time of year the students completed their pediatric clerkship. Scores were adjusted to the preceding academic year’s published standardizing norms by quarter: Quarter 1 percentiles for January-March, Quarter 2 for April-June, Quarter 3 for July-September, and Quarter 4 for October-December students. We compared these adjusted scores between two groups: pre-COVID-19 (students who rotated through the clerkship June 2017-February 2020) and during COVID-19 pandemic (students who rotated through the clerkship March-December 2020). Students with adjusted scores less than the fifth percentile nationally were considered to have failed their NBME shelf exam per IUSOM’s elected passing score cutoffs. Groups were compared with chi-square of independence testing and one-way analysis of variance (ANOVA) as detailed in the “Results” section. Statistical analysis was completed using Microsoft Excel Real Statistics Add-In (Microsoft Corporation, Redmond, Washington). Institutional Review Board approval (Protocol #2005735922, informed consent waived) was obtained prior to initiation of this study.

## Results

Of the 991 students at IUSOM who completed their pediatric shelf exam during the study period, 38 (3.8%) failed the exam on their first attempt. Table 1 shows pass/fail breakdowns by year, grouped into pre-pandemic and pandemic groups. Students taking the exam during the COVID-19 pandemic (March-December 2020) were more likely to fail than those taking it prior to the COVID-19 pandemic (8.7% vs. 2.5%, *X*^2^ = 17.87, *p*< 0.001). Students who completed their pediatric clerkship during the COVID-19 pandemic (March-December 2020) were 3.77 times more likely to fail their end-of-clerkship NBME shelf exam compared to those who completed the clerkship pre-pandemic (*p* < 0.001). A one-way ANOVA demonstrated that there was no significant difference in adjusted scores between years (*F*(3,987) = 0.29, *p* = 0.83). There was also no significant difference between adjusted scores in the pre-pandemic vs. pandemic groups (*F*(1,989) = 0.53, *p* = 0.47). Table 2 shows further shelf exam score breakdowns and analyses by quarter. One-way ANOVA comparing adjusted scores by quarter still demonstrated no significant difference, *F*(4,261) = 1.03, *p* = 0.39. Chi-square of independence testing revealed no significant difference between shelf exam failure rates when broken down by quarter, *X*^2^ = 4.58, *p* = 0.33.

**Table 1:** Breakdown of NBME corrected score reports by year and grouping pre-pandemic vs. pandemic. NBME: National Board of Medical Examiners.

	Pre-Pandemic				Pandemic	Statistical Analyses	
Year	2017	2018	2019	2020	2020	--	Total
# Passed	141	306	261	45	200	*X*^2^= 17.87, *p* < 0.001	953
# Failed	2	9	6	2	19		38
Mean NBME Corrected Score	54.1	51.5	52.5	52.5	50.9	*F*(1,989) = 0.53, *p* = 0.47	52.2
Median NBME Corrected Score	59	52	54	53	53	--	53
Total	143	315	267	47	219	--	991

**Table 2:** Breakdown of annual NBME corrected score reports by quarter. IQR: interquartile range; NBME: National Board of Medical Examiners.

		Pre-Pandemic				Pandemic	Statistical Analyses
Quarter		2017	2018	2019	2020	2020	*X*^2^= 4.58, *p* = 0.33 *F*(4,261) = 1.03, *p* = 0.39
1	# Passed	73	57	65	--	63	
	# Failed	1	1	0		8	
	Mean	57.6	54	54.0		48.1	
	Median IQR (1, 3)	63 (29, 79)	53 (36, 78)	55 (31, 78)		54 (23.5, 72)	
2	# Passed	68	74	66	--	55	
	# Failed	1	3	2		7	
	Mean	50.3	55.6	59.0		54.4	
	Median IQR (1, 3)	55 (22, 77)	57 (30, 79)	67 (39, 87)		53 (30.75, 88)	
3	# Passed	--	103	67	22	37	
	# Failed		2	3	1	1	
	Mean		50.4	45.5	57.2	45.4	
	Median IQR (1, 3)		49 (27, 72)	47.5 (21, 67)	62 (43, 82.5)	41 (14.25, 77)	
4	# Passed	--	72	63	23	45	
	# Failed		3	1	1	3	
	Mean		47.2	51.6	52.6	55.1	
	Median IQR (1, 3)		47 (18, 67)	48 (26, 71.25)	58 (25.25, 72.25)	60 (41, 72.25)	
	Total	143	315	267	47	219	

## Discussion

We found that students who completed their core pediatric clerkship during the COVID-19 pandemic were significantly more likely to fail their end-of-clerkship NBME shelf exam. Even after IUSOM students were re-introduced to the clinical setting in August of 2020, failure rates did not significantly improve. There are several potential reasons that may explain these findings. First, students who have not studied for Step 1 may not be prepared for this caliber of a standardized exam. Along the same lines, students may have been simultaneously studying for both the USMLE Step 1 and NBME shelf exams, perhaps not allocating enough attention to either exam. Second, students had less clinical exposure due to decreased quantity of pediatric patients seeking care in addition to restrictions placed regarding interaction with potential and confirmed COVID-19 patients [5]. This overall lack of exposure may have created difficulties in applying the information for some students, leading to poor performance on the shelf exam. Finally, the stress and burnout brought on/worsened by the pandemic and the transition to more virtual learning platforms have been associated with significant mental health deterioration, which likely contributed to the increase in shelf exam failure rates [9,10].

The COVID-19 pandemic has disrupted core clerkships for third-year medical students, forcing institutions to transition to virtual curriculums and adapt the role of the medical student in clinical settings to optimize safety measures for all. There have been quite a few studies evaluating the implications of pandemic-related abrupt alterations to traditional medical student curricula in surgery, but the literature regarding pediatrics curricula is more sparse [11,12]. Social distancing, mask wearing, hand washing, and class suspension definitely prompted a rapid decrease in the more common pediatric infectious diagnoses such as upper respiratory infections, hand-foot-mouth disease, and viral/bacterial pneumonias [3,5]. Not only did overall incidence of these illnesses decrease, but medical student involvement in the now much more limited care opportunities also decreased due to personal protective equipment conservation efforts [3]. Korean medical schools took a similar approach to US schools and on a similar timeline in terms of how they went about removing and later re-introducing their students to the pediatric clinical settings [5]. A Korean study noted that opportunities to be a part of counseling regarding normal development and routine vaccination schedules were nearly impossible once primary care clinics closed and went virtual [5]. Once permitted back on rotations in person, the limiting of social interactions and social distancing overall continued to impact medical student education. For example, less students could participate in inpatient rounds at any one time [5]. This, combined with increased parent refusal of medical student involvement in their children’s care, continued to limit learning opportunities [5]. While our study focused on the potential implications of reduced pediatric patient contact time/experiences during the COVID-19 pandemic on third-year medical student shelf exam scores, it is important to think about how they are also impacting their total medical education and limiting their clinical skill development in areas such as patient interviewing, physical examinations, and clinical decision-making as these medical students eventually apply for (and become employed by) residency programs [3,4,6].

While the times may seem unprecedented right now, similar findings to our study were demonstrated in Tulane University School of Medicine students’ standardized examination scores after the school’s temporary closure during/post-Hurricane Katrina in 2005 [6,13]. Students’ post-Katrina national standardized examination grades significantly decreased compared to pre-Katrina exam grades across many specialties, including Pediatrics (*p* < 0.05) [13]. Interestingly, however, there was no statistical change in USMLE board scores [13]. The decline in shelf exam scores was attributed to external stressors, which are likely also at play during the ongoing COVID-19 pandemic [6,13]. These findings are similar to our study, which found that students who completed their pediatric clerkship in pandemic times were much more likely to fail their end-of-clerkship shelf exam compared to those who completed the clerkship before pandemic, despite the fact that there were not significant differences between annual shelf exam mean and median scores. The significance of external stressors is also supported by the fact that even as the pandemic persisted and medical students were gradually re-integrated back into clinical settings, there was no statistically significant improvement in shelf exam failure rates. Like Hurricane Katrina, COVID-19 has disproportionately affected certain regions of the country [6], so relying on standardized test scores to compare medical student performance nationwide may not be as uniform or objective as it has been in the past [6]. Something to consider is that NBME test scores often have significant bearing on clerkship grades, which factor into residency applications [6].

The implementation of new learning and assessment strategies during the pandemic has been rushed and is not without flaws [1,6], as supported by our study and the lack of improvement in failure rates despite development of a more hybrid/in-person clinical clerkship experience as the pandemic continued. However, these unprecedented times provide an opportunity to “broaden the [medical trainee] assessment toolbox” [1]. Institutions have started to develop workplace-based assessment tools, remote assessment through oral examinations, and standardized patient telehealth visits [1,4,5]. These non-traditional evaluation methods may be more appropriate to assess medical student learning during non-traditional times.

Despite these important findings, our study has several limitations. Due to the study design, reasons for shelf exam failure rate outside of timing of the clerkship before or during pandemic were not able to be identified. Additionally, no individual student level data were available, limiting further assessment of the reasons that students may have failed their NBME shelf exam. Finally, other standardized test results such as USLME Step 1 scores were not available for comparison in this study.

## Conclusions

The COVID-19 pandemic increased medical student pediatric shelf exam failure rates at our large institution. While initial failure rate increases may be attributed to the rapid transition to a virtual learning platform, failure rates did not improve once students were re-integrated back into clinical settings. These findings suggest issues with acquisition and/or retainment of pediatric knowledge throughout the clerkship, which may be indicative of knowledge gaps in students’ pediatric education foundation secondary to the lack of requiring passing USMLE Step 1 prior to the initiation of clinical clerkships, the modified virtual/hybrid pediatric clerkship rotation in the context of the ever-evolving pandemic, the external stressors and burnout accompanying the still ongoing pandemic, or a combination of some/all of these issues. Trouble-shooting each of these potential contributors to medical student shelf exam failure rates is of utmost importance to ensure we are continuing to prioritize medical student learning and success. Further analyses of effects of the coronavirus pandemic on clinical skills are needed to better understand long-term implications of the COVID-19 pandemic on medical student education.
